# Disturbed phospholipid homeostasis in endoplasmic reticulum initiates tri-*o*-cresyl phosphate-induced delayed neurotoxicity

**DOI:** 10.1038/srep37574

**Published:** 2016-11-24

**Authors:** Li Zhu, Pan Wang, Ying-Jian Sun, Ming-Yuan Xu, Yi-Jun Wu

**Affiliations:** 1 Laboratory of Molecular Toxicology, State Key Laboratory of Integrated Management of Pest Insects and Rodents, Institute of Zoology, Chinese Academy of Sciences, Beijing 100101, China; 2 University of Chinese Academy of Sciences, Beijing 100049, China; 3 Department of Veterinary Medicine and Animal Science, Beijing Agriculture College, Beijing 102206, China

## Abstract

Tri-*o*-cresyl phosphate (TOCP) is a widely used organophosphorus compound, which can cause a neurodegenerative disorder, i.e., organophosphate-induced delayed neurotoxicity (OPIDN). The biochemical events in the initiation of OPIDN were not fully understood except for the essential inhibition of neuropathy target esterase (NTE). NTE, located in endoplasmic reticulum (ER), catalyzes the deacylation of phosphatidylcholine (PC) and lysophosphatidylcholine (LPC) to glycerophosphocholine (GPC). The present study aims to study the changes of ER phospholipids profile as well as levels of important intermediates of phospholipid synthesis such as diacylglycerol (DAG) and phosphatidic acid (PA) at the initiation stage of OPIDN. Hens are the most commonly used animal models of OPIDN. The spinal cord phospholipidomic profiles of hens treated by TOCP were studied by using HPLC-MS-MS. The results revealed that TOCP induced an increase of PC, LPC, and sphingomyelin (SM) levels and a decrease of GPC, phosphatidylethanolamine (PE), lysophosphatidylethanolamine (LPE), lysophosphatidylserine (LPS), phosphatidylglycerol (PG), and phosphatidylinositol (PI) levels., Levels of DAG and PA were also decreased. Pretreatment with phenylmethylsulfonyl fluoride (PMSF) 24 h before TOCP administration prevented OPIDN and restored the TOCP-induced changes of phospholipids except GPC. Thus, the disruption of ER phospholipid homeostasis may contribute to the initiation of organophosphate-induced delayed neurotoxicity.

Organophosphorous compounds (OP) have been widely used in agriculture, industry and household. However, reports of poisoning by exposing to these compounds continue to appear^1^,^2^. The main symptoms of OP poisoning include acute cholinergic syndrome, intermediate syndrome and OP-induced delayed neurotoxicity (OPIDN)^3^,^4^. Certain OPs such as tri-*o*-cresyl phosphate (TOCP), which is used mainly in industry as a lubricating oil additive or a softener, are able to induce OPIDN. The clinical symptoms of OPIDN occur 1–3 weeks following exposure to OPs. OPIDN is a neurodegenerative disorder characterized by ataxia, which progresses to paralysis with a concomitant distal degeneration of some long and large-diameter axons in the peripheral nerves and the spinal cord^5^. Hens are the most sensitive animal model of OPIDN and the clinical signs were similar to those seen in humans^6^.

The putative molecular target of OP responsible for the initiation of OPIDN is neuropathy target esterase (NTE), which is a member of the serine hydrolase family protein^7^. OPIDN requires more than 70% inhibition of NTE activity within 1–2 days post-dosing. In addition, aging of NTE, which involves increased negative charge at the active site, is also required for OPIDN^8^,^9^. Studies have shown that NTE is anchored to the cytoplasmic face of the endoplasmic reticulum (ER), where it catalyzes the deacylation of phosphatidylcholine (PC) and lysophosphatidylcholine (LPC) to glycerophosphocholine (GPC)^10^,^11^,^12^. The biochemical function of NTE indicates that inhibition of NTE by OP may, at least temporarily, disrupt phospholipid homeostasis in ER. However, the detailed underlying molecular mechanism of OPIDN is not completely understood.

Phospholipid bilayers maintain the structure and functionality of all unicellular and multicellular membranes. ER is the primary site for phospholipid synthesis and contains >60% of phospholipid mass in a variety of cell types^13^. The phospholipid content and composition in ER are in constant changes triggered by different cellular events^13^,^14^,^15^. Phospholipid imbalances can lead to many neurological disorders, including bipolar disorders, schizophrenia, and neurodegenerative diseases such as Alzheimer’s, Parkinson’s, Niemann-Pick and Huntington diseases^16^,^17^.

In eukaryotic cells, ER is the starting point for all secretory transport, which is important for axonal transport as well as the interaction between the axon and supporting glial cells^18^. Inhibition of the retrograde axonal transport has been demonstrated in the OP-dosed chickens before the onset of ataxia^19^. And abnormal glial-axonal/neuronal interaction was the earliest detected pathological change in the brains of *swiss cheese* (homolog of NTE in Drosophila) mutant flies^20^. Thus disruption of ER’s secretory function may play an important role in the initiation of OPIDN. Since ER phospholipid imbalances may cause malfunctions of the ER, ER phospholipid imbalances may be the causal link between OP-modified NTE and axonal degeneration in OPIDN.

Phenylmethylsulfonyl fluoride (PMSF) is a non-neuropathic NTE inhibitor, which inhibits the activity of NTE but does not produce aged NTE. It has been reported that hens pretreated with PMSF were completely protected against neuropathy from subsequently administered neuropathic OP inhibitors such as TOCP^21^. Phospholipidomics is a powerful research tool that can be utilized to investigate phospholipid pathways, which play important roles in cell biology and in specific disease processes. Phospholipidomics approaches have therefore been used in cultured cells, in animal studies and increasingly in the human pathophysiological context^16^,^17^. Thus, in the current study, to investigate the relationship between ER phospholipid profile and OPIDN, phospholipidomics was employed to characterize ER phospholipid profiles in hens exposed to TOCP with or without pretreatment with PMSF. To our knowledge, this is the first phospholipidomics analysis for OPIDN.

## Results

### Clinical signs and NTE activity

Signs of delayed neurotoxicity were first observed on day 7 post-dosing in hens treated with TOCP (mean score = 1.0 ± 0.17). These hens developed complete paralysis by day 21 (mean score = 7.8 ± 0.17). However, no clinical signs of delayed neurotoxicity were observed for hens that were pretreated with PMSF for 24 h and then treated with TOCP during the whole experiment period. Thus, pretreatment with PMSF before the administration of TOCP protected the hens from the development of the delayed neurotoxicity.

NTE is the direct molecular target of TOCP. Compared to control group, NTE activity was reduced to 11% on day 2 after TOCP treatment. However, although PMSF pretreatment prevented the delayed neurotoxicity in hens induced by TOCP, NTE activity inhibition was not prevented by PMSF pretreatment compared to that by TOCP alone treatment (data not shown).

### Comparative phospholipidomics

**Figure 1:**
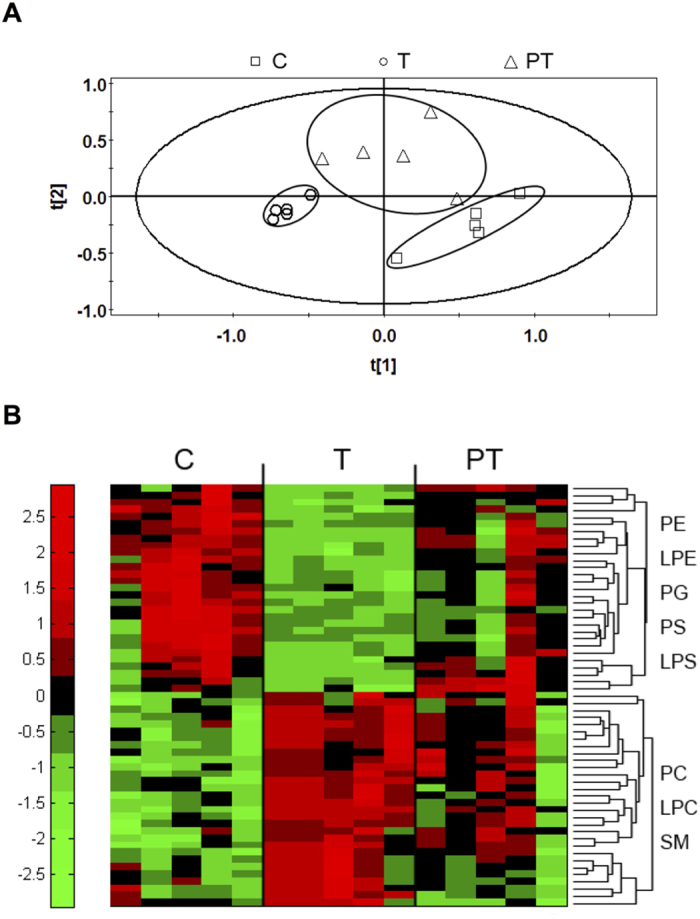
Phospholipidomic analysis of spinal cord ER phospholipids from hens. Adult hens were orally administrated with vehicle (empty capsule) (control, C), TOCP (750 mg/kg) (T), or TOCP (750 mg/kg) 24 h after PMSF pretreatment (60 mg/kg, s.c.) (PT), N = 5 in each test group. The spinal cord samples were collected on day 2 after TOCP administration, and then the phospholipids profile was detected by HPLC-ESI-MS/MS method. (**A**) Score plot of the multivariate PLS-DA on the ER phospholipid composition. Symbols: ◽, Control (**C**); ○, TOCP (T); **∆**, PMSF + TOCP (PT). (**B**) Heat map showing the levels of changed ER phospholipids in different treatments. Fifty-nine phospholipids with VIP values >1.0 and P < 0.05 were selected: 16 PCs, 5 LPCs, 9 SMs, 22 PEs, 4 LPEs, 1 PG, 1 PS, 1 LPS species. Each column represents values from an individual animal. Different colors indicate the levels of each phospholipid (Red indicates that the value is higher than the average and green lower than the average). Abbreviations: ER, endoplasmic reticulum; LPC, lysophosphatidylcholine; LPE, lysophosphatidylethanolamine; LPS, lysophosphatidylserine; PLS-DA, partial least square-discriminant analysis; PC, phosphatidylcholine; PE, phosphatidylethanolamine; PG, phosphatidylglycerol; PS, phosphatidylserine; SM, sphingomyelin; TOCP, tri-*o*-cresyl phosphate.

To study the changes of ER phospholipid homeostasis induced by TOCP, phospholipidomics analyses were performed using the ER fraction from spinal cord samples of hens in control, TOCP, and PMSF plus TOCP groups. Total 201 phospholipid species from 9 classes, i.e., phosphatidylcholine (PC), lysophosphatidylcholine (LPC), phosphatidylethanolamine (PE), lysophosphatidylethanolamine (LPE), phosphatidylserine (PS), lysophosphatidylserine (LPS), phosphatidylglycerol (PG), phosphatidylinositol (PI), and sphingomyelin (SM) were identified. Partial least square-discriminant analysis (PLS-DA) was carried out for the phospholipid composition of spinal cord ER in control, TOCP, and PMSF plus TOCP groups. As shown in Fig. 1, the PLS-DA plot showed that the data points in control group were clearly separated from those in TOCP group. Interestingly, Data points in PMSF pretreatment group (PT) located between those in control group and TOCP groups (Fig. 1A).

**Figure 2:**
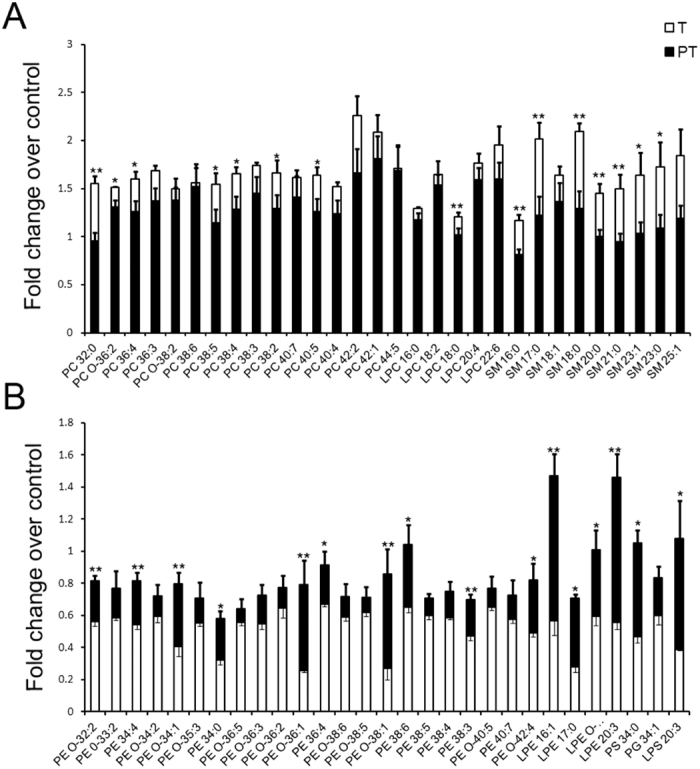
The levels of each changed phospholipid in different treatments. Adult hens were orally administrated with vehicle (empty capsule) (control, C), TOCP (T), or TOCP 24 h after PMSF pretreatment (PT). The spinal cord samples were collected on day 2 after TOCP administration. Phospholipids profile was detected by HPLC-ESI-MS/MS method. The phospholipids were increased (**A**) or decreased (**B**) in TOCP group (white bars) compared to control. Black bars indicate levels of phospholipids in PT group. Data are normalized to those in control group and are expressed as fold change compared to control group. All the phospholipid species were significantly changed in TOCP group compared to control group with P values < 0.05 (n = 5). Data were expressed as mean ± SEM (n = 5). *P < 0.05, and **P < 0.01, compared to TOCP group (n = 5).

Next, the phospholipids contributing most to the separation of these three groups according to variable importance plot (VIP) values were identified. VIP is a weighed sum of squares of the PLS weight, and VIP values indicate the importance of the variables to the whole model. Fifty-nine phospholipids with VIP values larger than 1.00 and P values less than 0.05 were identified to have significant different levels among the three groups (Fig. 1B). Compared to control, 30 out of 59 phospholipids were increased in TOCP group, which belong to 3 classes: PC (16 phospholipids), LPC (5 phospholipids) and SM (9 phospholipids) (Fig. 2A, white bars). The other 29 phospholipids were decreased in TOCP group, which belong to 5 classes: PE, LPE, PG, PS and LPS. Most of these 29 phospholipids were PE and LPE species (22 and 4, respectively) (Fig. 2B, white bars). Interestingly, levels of all the 59 phospholipids were restored, at least partly, in PMSF plus TOCP group (Fig. 2, black bars).

**Figure 3:**
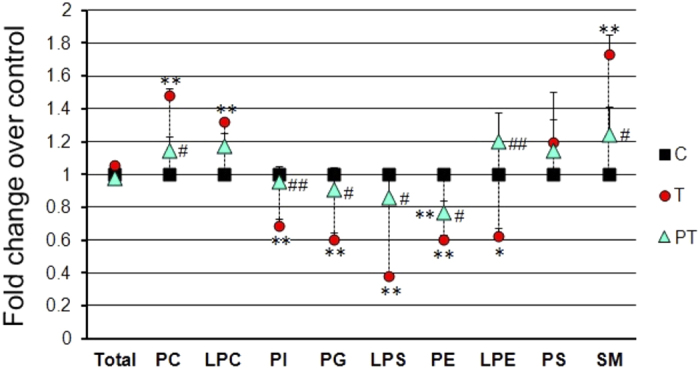
The levels of phospholipids in different classes in the three groups. Adult hens were orally administrated with vehicle (control, C), TOCP (T), or TOCP 24 h after PMSF pretreatment (PT). The spinal cord samples were collected on day 2 after TOCP administration. Phospholipids profile was detected by HPLC-ESI-MS/MS method. Data are normalized to those of control group and are expressed as fold change compared to control group. Data were expressed as mean ± SEM (n = 5). *P < 0.05, and **P < 0.01, compared to the control group; ^#^P < 0.05 and ^##^P < 0.01, compared to TOCP group.

Furthermore, the total phospholipids in each individual lipid class in these three groups were compared. Figure 3 showed that compared to control, TOCP induced a prominent increase of PC, LPC, and SM and an obvious decrease of PE, PI, PG, LPE, and LPS. PS levels did not change after TOCP administration. Interestingly, total phospholipids levels were not altered by TOCP treatment. Surprisingly, although NTE inhibition by TOCP treatment was similar by PMSF pretreatment, it reversed the increase of PC, LPC, and SM as well as the decrease of PE, PG, LPS, PI, and LPE induced by TOCP.

### Effect of PMSF pretreatment on recovery of GPC level

**Figure 4:**
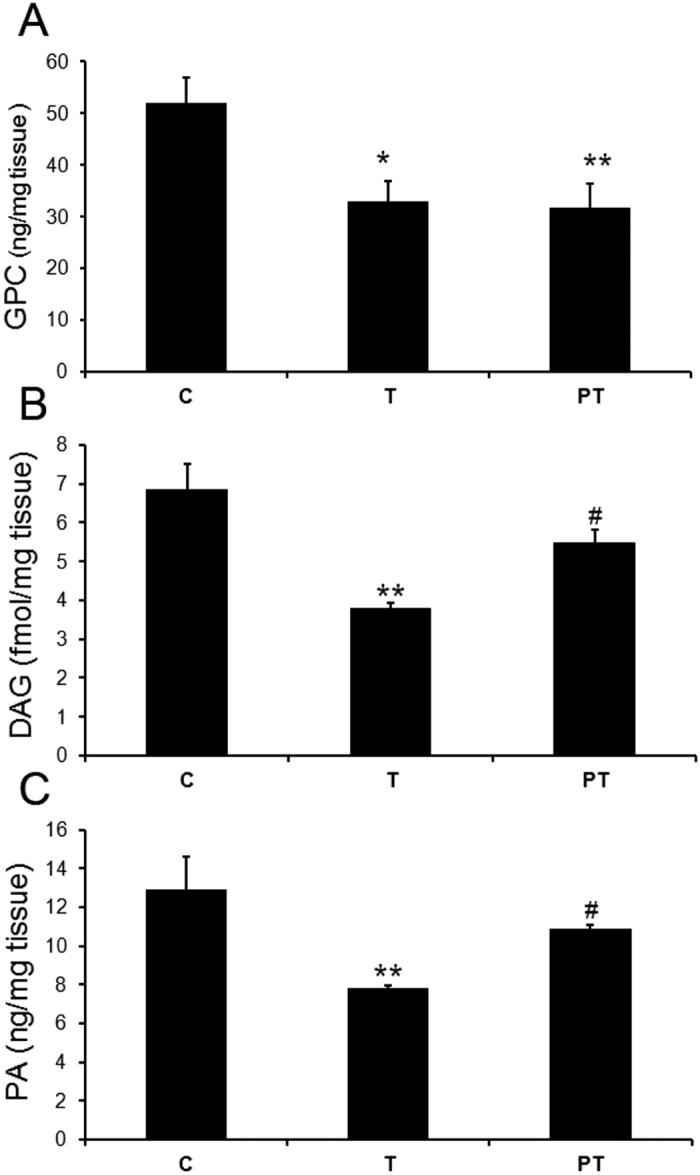
Effect of TOCP and PMSF on the GPC (**A**), DAG (**B**) and PA (**C**) levels in spinal cord of hens. Adult hens were orally administrated with vehicle (control, C), TOCP (T), or TOCP 24 h after PMSF pretreatment (PT). The spinal cord samples were collected on day 2 after TOCP administration. GPC content was detected by HPLC-ESI-MS and DAG and PA levels were measured by ELISA assay. GPC and PA concentrations were expressed as ng per mg of spinal cord sample. DAG concentrations were expressed as fmol per mg of spinal cord sample. Data were expressed as mean ± SEM (n = 5). *P < 0.05, and **P < 0.01, compared to the control group. ^#^P < 0.05, compared to TOCP group.

NTE acts as phospholipase B and catalyzes the deacylation of PC and LPC to GPC. GPC levels in spinal cord were further measured by HPLC-ESI-MS. There was a statistically significant decrease (decreased to 63% of control) of GPC content in TOCP treatment hens (T group). Meanwhile, PMSF pretreatment (PT group) also decreased the GPC content (decreased to 61% of control) (Fig. 4A), which indicated PMSF did not restore the NTE activity inhibited by PMSF, and the decrease of GPC level was not directly related with OPIDN initiation.

### Effect of PMSF pretreatment on recovery of DAG and PA levels

Diacylglycerol (DAG) and phosphatidic acid (PA) are important intermediate metabolites in phospholipids homeostasis, which serve as both the substrates of phospholipids synthesis and products of phospholipids degradation. Thus DAG and PA levels in spinal cord ER were measured using ELISA. The result showed that PA and DAG levels in TOCP alone treated hens were significantly decreased compared to control, and PMSF pretreatment could markedly protected the reduction of PA and DAG levels (Fig. 4B and C).

## Discussion

OPIDN is a neurodegenerative condition that specifically affects nerves with long axons in both the central and peripheral nervous systems. In this study, OPIDN was successfully induced in hens using a single dose of TOCP and pretreatment with PMSF prevented hens from OPIDN. Our phospholipidomics analysis showed that pretreatment with PMSF restored the TOCP-induced changes of phospholipids except GPC in the early phase of OPIDN. Thus this study suggests that disruption of ER phospholipid homeostasis may play an important role in the initiation development of OPIDN.

NTE is thought to be the initiation site of OPIDN and inhibition of NTE is necessary but not sufficient for OPIDN initiation (Johnson 1982). Consistently, our data showed that NTE was significantly inhibited (>85%) on day 2 in both TOCP treatment and PMSF pretreatment hens. NTE is a member of a 9-protein family of patatin-like phospholipase domain-containing proteins (PNPLAs), of which NTE is PNPLA6^18^. It is anchored to ER membrane and catalyzes the deacylation of PC and LPC to GPC. This suggests that disruption of ER phospholipid homeostasis may be implicated in OPIDN.

Phosphatidylcholine (PC) is the most abundant phospholipid in eukaryotic cell membranes, and disruption of the PC homeostasis was found in many neurodegenerative disorders^22^. LPC is the product of PC degradation by phospholipase A (PLA). An elevated LPC level can induce neuronal sheath demyelination and a variable degree of axonal degeneration^23^,^24^, which is similar to the pathological changes of OPIDN. As expected, the PC and LPC levels were remarkably elevated and GPC level was significantly decreased 2 d after TOCP administration, which indicated that inhibition of NTE by TOCP disturbed the balance among PC, LPC and GPC. The present result is consistent with the findings in mice^25^. However, our previous study showed that no changes of PC and LPC were observed after TOCP exposure in hens using one-dimensional TLC^26^. The detection limit of the method and the time point chosen to study may be the reasons that cause the difference.

**Figure 5:**
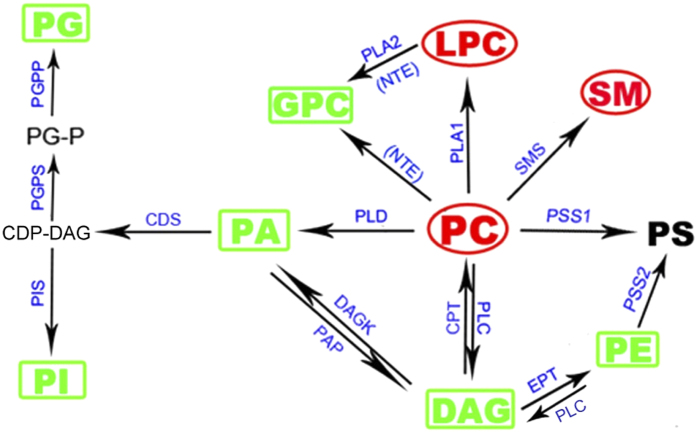
Summary of the change of phospholipids involved in PC biosynthesis, degradation and conversion in spinal cord ER after TOCP treatment. Adult hens were orally administrated with vehicle (control, C), TOCP (T), or TOCP 24 h after PMSF pretreatment (PT). The spinal cord samples were collected on day 2 after TOCP administration. The key metabolites in phospholipid biosynthesis and the enzymes catalyzing the respective reactions are indicated. NTE was inhibited (in the bracket). Some phospholipids increased after TOCP administration (in red ellipse); while other phospholipids decreased by TOCP (in green box). All of the changed phospholipids except for GPC were restored by the PMSF pretreatment. Abbreviations: CDP: cytidine diphosphate; CDP-DAG, CDP-diacylglycerol; CDS, CDP-DAG synthase; CPT, 1,2-diacylglycerol cholinephosphotransferase; DAG, diacylglycerol; DAGK, DAG kinase; EPT, CDP-ethanolamine:diacylglycerol ethanolaminephosphotransferase; GPC, glycerophosphocholine; LPC, lysophosphatidylcholine; NTE, neuropathy target esterase; PA, phosphatidic acid; PAP, PA phosphatases; PC, phosphatidylcholine; PE, phosphatidylethanolamine; PG, phosphatidylglycerol; PGP, PG phosphate; PGPS, PGP synthase; PGPP, PGP phosphatase; PI, phosphatidylinositol; PIS, PI synthase; PLA1, phospholipase A1; PLA2, phospholipase A2; PLC, phospholipase C; PLD, phospholipase D; PSS1, phosphatidylserine synthase 1; PSS2, phosphatidylserine synthase 2; SM, sphingomyelin; SMS, SM synthase.

Our result showed that total phospholipids were not affected by TOCP treatment. Hence the increase of PC was accompanied by the decrease of other phospholipids. PE, synthesized via the Kennedy pathway by using ethanolamine and DAG in ER, was remarkably decreased. PG and PI, synthesized by a common precursor PA, were both decreased. The conversion of DAG to PA could contribute to the balance of phospholipid synthesis between the pathway producing PC, PE and PS and that producing PI and PG^27^. DAG and PA, substrates of phospholipids synthesis and products of phospholipids degradation, were also significantly decreased after TOCP treatment. No alternation was observed in PS, which is synthesized by base-exchange from PC or PE by PS synthases. However, SM, which is synthesized by PC and ceramide^28^, was significantly increased. These data suggested that TOCP interfered with ER phospholipid homeostasis through disturbing pathways of phospholipid biosynthesis and interconversion in ER (Fig. 5).

**Figure 6:**
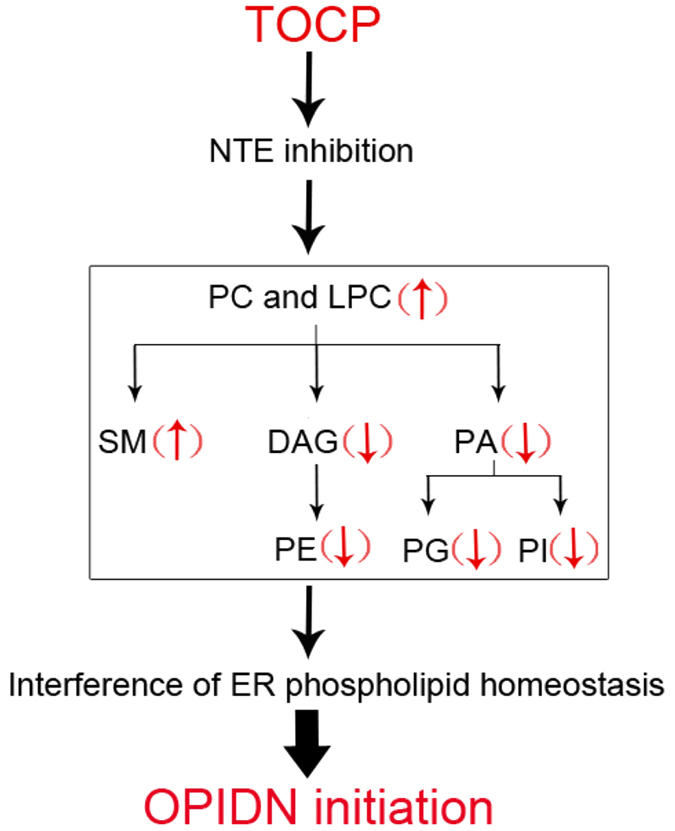
Schematic diagram for the hypothesis on the pathways involved in OPIDN initiation. Arrows indicated the changes of phospholipids upon TOCP treatment. Abbreviations: DAG, diacylglycerol; GPC, glycerophosphocholine; LPC, lysophosphatidylcholine; NTE, neuropathy target esterase; PA, phosphatidic acid; PC, phosphatidylcholine; PE, phosphatidylethanolamine; PG, phosphatidylglycerol; PI, phosphatidylinositol; SM, sphingomyelin.

Surprisingly, although GPC level in PMSF plus TOCP-treated hens was similar as that in TOCP alone treated hens, PC and LPC levels were decreased in PMSF plus TOCP-treated hens compared to TOCP alone treated hens. This result suggested that the alteration of GPC level was not responsible for OPIDN initiation. Meanwhile, DAG, PA and other changed phospholipids were all at least partly restored in PMSF-pretreated hens compared to TOCP alone treated hens. Thus we proposed that increase of PC induced by TOCP may lead to an increase of SM and a decrease of DAG and PA, which then lead to reduced synthesis of PE, PG, and PI (Fig. 6). However, since the maintenance of ER phospholipid homeostasis is highly complicated, the detailed mechanism merits further investigation.

It is of note that aging of NTE is discovered to be important in OPIDN initiation^8^,^9^. Pretreatment with protease inhibitor PMSF has been shown to protect the aging of NTE and prevent the development of delayed symptoms in hens^29^. Thus the aging of NTE correlated with the results of the present study, i.e., PMSF reversed most of the phospholipid changes induced by TOCP. However, the relationship between NTE aging and ER phospholipid homeostasis and how PMSF pretreatment regulates phospholipid homeostasis needs to be further investigated.

In summary, we revealed that the ER phospholipid homeostasis was disturbed due to NTE inhibition at the initiation stage of OPIDN. This result could contribute to the better understanding of the pathogenesis involved in OPIDN and may aid in the possible discovery of novel treatment for OPIDN.

## Materials and Methods

### Reagents

TOCP (purity >99%) was purchased from BDH Chemicals Co. Ltd. (Poole, England). Phenylmethylsulfonyl fluoride (PMSF) was purchased from Fluka Chemika (Buchs, Switzerland). Mipafox and phenyl valerate were synthesized in our laboratory as described previously^25^. 4-aminoantipyrine was obtained from Beijing Xizhong Chemical Factory (Beijing, China). Glycerophosphocholine (GPC), paraoxon, ammonium formate and formic acid were obtained from Sigma-Aldrich (St. Louis, MO, USA). The following synthetic lipid standards were purchased from Avanti Polar Lipids (Alabaster, AL, USA): 1,2-dimyristoyl-sn-glycero-3-phospho-(1′-rac-glycerol) (sodium salt) [PG(14:0/14:0)]; 1,2-dimyristoyl-sn-glycero-3-phosphoethanolamine [PE(14:0/14:0)]; 1,2-dimyristelaidoyl-sn-glycero-3-phosphocholine [PC(14:1/14:1)]; 1-(10Z-heptadecenoyl)-sn-glycero-3-phospho-(1′-rac-glycerol) (sodium salt) [LPG(17:1)]; and 1-(10Z-heptadecenoyl)-sn-glycero-3-phosphoethanolamine [LPE(17:1)]. N-hexane, isopropanol, methanol and chloroform of HPLC grade were obtained from CNW Technology (Shanghai, China). ELISA kits for chicken DAG and PA were obtained from Shanghai Guyan Industrial Co. Ltd. (Shanghai, China).

### Animals

Adult Beijing white laying hens (10 month, weighing 1.0–1.5 kg) were obtained from Dabei Poultry Farm (Beijing, China). They were housed two per cage in a temperature and humidity-controlled room (22 °C and 50% humidity), with a light/dark cycle of 12 h. The hens were acclimatized for 7 d prior to the start of the experiment.

All animal procedures were performed in accordance with the applicable Chinese legislation and approved by the Animal and Medical Ethics Committee of the Institute of Zoology, Chinese Academy of Sciences.

### Drug administration

Twenty-four hens were randomly divided into control group, TOCP group and PMSF plus TOCP group. The hens in TOCP group and PMSF plus TOCP group were orally given a single dose of TOCP with 750 mg/kg body weight in gelatin capsules. PMSF pretreatment hens were injected subcutaneously (s.c.) with PMSF (60 mg/kg) dissolved in dimethylsulfoxide (DMSO) and 24 h before TOCP administration. The control group hens were given empty gelatin capsules. Hens were examined daily for the delayed neurotoxic signs after the administration of TOCP until day 21. OPIDN neurological signs were assessed by an eight-point graded scale (0, normal ambulation; 1–2, slight and infrequent hind limb incoordination; 3–4, moderate but definite hind limb incoordination; 5–6, severe and frequent difficulty in walking and standing erect; 7–8, virtual to complete hind limb paralysis)^30^. Five hens in each group were sacrificed on day 2 after TOCP administration and the other 3 hens on day 21. The spinal cord samples were quickly dissected and frozen in liquid nitrogen and then kept at −80 °C for biochemical analysis.

### NTE activity assay

Spinal cord were homogenized in TE buffer (50 mM Tris–HCl, 0.2 mM EDTA, pH 8.0) and centrifuged at 100 g at 4 °C for 2 min. The supernatant fraction was used to determine NTE activity according to the procedure in the earlier report^31^. Briefly, 0.5 ml samples were incubated with 50 μM paraoxon and 0 or 25 μM mipafox at 37 °C for 20 min. Then 1 ml substrate solution (mixture of 1 volume of 15 mg/ml phenyl valerate and 30 volume of 0.03% Triton X-100 in TE buffer) was added to each of the reaction tubes. After incubation at 37 °C for 30 min, 1 ml stop buffer (3.33% SDS/0.025% 4-aminoantipyrine in TE buffer) was introduced and then 0.5 ml of 0.4% potassium ferricyanide in water was added. The absorbance was measured at 486 nm. NTE activity was calculated as the difference in phenol liberated from the paraoxon-resistant and mipafox-sensitive hydrolysis of phenyl valerate, and expressed as nanomoles of phenol formed per minute per milligram of protein with phenol as the standard. Concentrations of protein were measured by using the method of Bradford (1976) with bovine serum albumin as standard.

### Measurement of GPC levels

Spinal cord samples were homogenized in water with 0.1% formic acid and then chloroform with 0.1% formic acid was added to remove the proteins and lipids. The mixture was centrifuged at 13500 g at 4 °C for 20 min to separate the two phases. The upper phase was used to measure the GPC level using HPLC-electron spray ionization (ESI)-MS. The experiment was performed on an Agilent 1200 series HPLC system coupled to an Agilent 6520 Accurate-Mass Quadrupole Time-of-Flight mass spectrometry (QTOF MS) equipped with an Agilent ESI source (Agilent Technologies, CA, USA). Three microliter samples were injected onto a ZORBAX Extend-C18 column (2.1 × 50 mm, 1.8 μm, Agilent, US) with mobile phase flow rate of 0.2 ml/min. Mobile phase was 3% acetonitrile in water containing 0.1% formic acid and the elution lasted for 4 min. The HPLC column was maintained at 25 °C in a thermostat. The jet stream ESI source was operated in the positive mode. Instrument parameters were set as follows: sheath gas temperature, 350 °C; sheath gas flow, 10 L/min; nebulizer, 45 psi; dry gas temperature, 300 °C; dry gas flow, 5 L/min and capillary entrance voltage, 3500 V. Fragmentor and skimmer were operated at 175 V and 65 V, respectively. The MS scan data were collected at a rate of 1.02 spectra/s in the range of m/z 60–1100. The m/z of all ions in the mass spectra were corrected by two reference ions (m/z 121.050873 and 922.009798), which ensured that the mass error was less than 3 ppm throughout the experiment. GPC level was quantified according to the standard and normalized to the tissue weight.

### ER fractionation and lipid extraction

ER fractionation of spinal cord in hens were carried out as described by Cox and Emili^32^. Total lipids in ER were extracted by a modified Folch method^33^. Briefly, 0.5 g hen spinal cord samples were homogenized for 20 times in 1.5 ml STM buffer (0.25 M sucrose, 50 mM Tris pH 7.4, 5 mM MgCl_2_) by using a glass-homogenizer. The whole lysates were first cleared by centrifugation at 3000 g for 10 min, followed by centrifugation at 6000 g for 15 min to remove mitochondria. Then the supernatant was centrifuged at 100,000 g for 60 min to obtain ER pellet. The precipitant was re-suspended in 200 μl STM buffer. Lipids in ER suspensions were extracted by using 1 ml of chloroform-methanol (2:1 v/v) in the presence of appropriate amounts of internal standards [PG (14:0/14:0), PE (14:0/14:0), PC (14:1/14:1), LPG (17:1), LPE (17:1)] for phospholipids analyses. Samples were agitated with ultrasound for 15 min and then 200 μl of 0.25% KCl (w/v) was added to the samples. The samples were then mixed for 30 s and centrifuged at 13500 g at 4 °C for 20 min to separate the two phases. The lower organic phase was collected and dried by evaporation under a nitrogen stream. Then the lipid extracts were dissolved in 200 μl chloroform-methanol (2:1 v/v) and used for the phospholipid profile analysis and ELISA assay.

### ER phospholipid profile analysis

HPLC-ESI-MS/MS technique was used to analyze the phospholipid profile of the ER fraction of spinal cord as described previously^34^. Twenty microliter lipid samples were injected onto a Rx-SIL silica column (2.1 × 150 mm, 5 μm, Agilent, US) with mobile phase flow rate of 0.1 ml/min. Solvent A and B were hexane/isopropanol/water (30/70/2, v/v/v) and methanol/water (100/2, v/v), respectively, both containing 5 mM ammonium formate. The gradient elution began with 100% solvent A solution for 30 min and switched to 100% solvent B from 30.01 min to 62 min. The thermostat column compartment was operated at 25 °C. The jet stream ESI source was operated in the negative mode, and instrument parameters were set as follows: sheath gas temperature, 350 °C; sheath gas flow, 8 L/min; nebulizer, 20 psi; dry gas temperature, 300 °C; dry gas flow, 5 L/min; and capillary entrance voltage, 3500 V. Fragmentor and skimmer were operated at 190 V and 65 V, respectively. The MS scan data were collected at a rate of 1.02 spectra/s in the range of m/z 100–2000. The m/z of all ions in the mass spectra were corrected by two reference ions (m/z 112.985587 and 966.000725), which ensured that the mass error was less than 3 ppm throughout the experiment. MS data of phospholipids profiles were extracted by MassHunter Qualitative Analysis software (B.02.00). All detected lipids were quantified according to the corresponding internal standard and normalized to the tissue weight. Partial least square discriminant analysis (PLS-DA) was performed with SIMCA-P 11.5. The open-source MultiExperiment Viewer software was employed for heatmap generation.

### Enzyme-linked immunosorbent assay

DAG and PA contents in spinal cord ER were evaluated by enzyme-linked immunosorbent assay (ELISA). The experiments were carried out using commercially available kits according to the manufacturer’s protocols. Briefly, the microtiter plate provided in the kits was pre-coated with an antibody specific to chicken DAG or PA. Standards or samples were then added and incubated. DAG or PA antibody conjugated to horseradish peroxidase (HRP) was added and incubated. Then a TMB substrate solution was added to each well. The enzyme-substrate reaction was terminated by the addition of sulfuric acid solution and the color change was measured by a spectrophotometer at a wavelength of 450 nm. The concentrations of DAG or PA in the samples were then determined according to the standard curve.

### Statistical analysis

All values were presented as mean ± SEM from five animals. Statistically significant differences (P < 0.05 and P < 0.01) were determined by ANOVA using SPSS 12.0 statistical software. Comparisons of mean values were performed by the Tukey’s test.

## Additional Information

**How to cite this article**: Zhu, L. *et al*. Disturbed phospholipid homeostasis in endoplasmic reticulum initiates tri-*o*-cresyl phosphate-induced delayed neurotoxicity. *Sci. Rep.* **6**, 37574; doi: 10.1038/srep37574 (2016).

**Publisher’s note:** Springer Nature remains neutral with regard to jurisdictional claims in published maps and institutional affiliations.

## References

[b1] Chowdhary S., Bhattacharyya R. & Banerjee D. Acute organophosphorus poisoning. Clin. Chim. Acta. 431, 66–76 (2014). 24508992 10.1016/j.cca.2014.01.024

[b2] Sarkar S., Nandi M., Mondal R. & Mandal S. K. Organophosphorus-induced extrapyramidal intermediate syndrome in an adolescent suicide attempt survivor. J. Neurosci. Rural Pract. 5(3), 276–278 (2014). 25002771 10.4103/0976-3147.133596 PMC4078616

[b3] Paudyal B. P. Organophosphorus Poisoning. J. Nepal. Med. Assoc. 47(4), 251–258 (2008). 19079407

[b4] Hargreaves A. J. Neurodegenerations induced by organophosphorous compounds. Adv. Exp. Med. Biol. 724, 189–204 (2012). 22411244 10.1007/978-1-4614-0653-2_15

[b5] Johnson M. K. Symposium introduction - Retrospect and prospects for neuropathy target esterase (NTE) and the delayed polyneuropathy (OPIDP) induced by some organophosphorus esters. Chem. Biol. Interact. 87 **(1–3)**, 339–346 (1993). 8343991 10.1016/0009-2797(93)90062-4

[b6] Lotti M. & Johnson M. K. Neurotoxicity of organophosphorus pesticides: predictions can be based on *in vitro* studies with hen and human enzymes. Arch. Toxicol. 41(3), 215–221 (1978). 736792 10.1007/BF00354093

[b7] Johnson M. K. The target for initiation of delayed neurotoxicity by organophosphorus esters: biochemical studies and toxicological applications. Rev. Biochem. Toxicol. 4, 141–212 (1982).

[b8] Richardson R. J., Hein N. D., Wijeyesakere S. J., Fink J. K. & Makhaeva G. F. Neuropathy target esterase (NTE): overview and future. Chem. Biol. Interact. 203(1), 238–244 (2013). 23220002 10.1016/j.cbi.2012.10.024

[b9] Johnson M. K. Organophosphates and delayed neuropathy–is NTE alive and well? Toxicol. Appl. Pharmacol. 102(3), 385–399 (1990). 2180130 10.1016/0041-008x(90)90036-t

[b10] Li Y., Dinsdale D. & Glynn P. Protein domains, catalytic activity, and subcellular distribution of neuropathy target esterase in mammalian cells. J. Biol. Chem. 278(10), 8820–8825 (2003). 12514188 10.1074/jbc.M210743200

[b11] Quistad G. B., Barlow C., Winrow C. J., Sparks S. E. & Casida J. E. Evidence that mouse brain neuropathy target esterase is a lysophospholipase. Proc. Natl. Acad. Sci. USA 100(13), 7983–7987 (2003). 12805562 10.1073/pnas.1232473100 PMC164699

[b12] Zaccheo O., Dinsdale D., Meacock P. A. & Glynn P. Neuropathy target esterase and its yeast homologue degrade phosphatidylcholine to glycerophosphocholine in living cells. J. Biol. Chem. 279(23), 24024–24033 (2004). 15044461 10.1074/jbc.M400830200

[b13] Lagace T. A. & Ridgway N. D. The role of phospholipids in the biological activity and structure of the endoplasmic reticulum. BBA-Mol. Cell Res. 1833(11), 2499–2510 (2013). 10.1016/j.bbamcr.2013.05.018 23711956

[b14] Fagone P. & Jackowski S. Membrane phospholipid synthesis and endoplasmic reticulum function. J. Lipid Res. 50 (Suppl), S311–S316 (2009). 18952570 10.1194/jlr.R800049-JLR200 PMC2674712

[b15] Vance J. E. Phospholipid Synthesis and Transport in Mammalian Cells. Traffic. 16(1), 1–18 (2015). 25243850 10.1111/tra.12230

[b16] Adibhatla R. M. & Hatcher J. F. Role of lipids in brain injury and diseases. Future lipidol. 2(4), 403–422 (2007). 18176634 10.2217/17460875.2.4.403 PMC2174836

[b17] Cheng D. . Lipid pathway alterations in Parkinson’s disease primary visual cortex. PLoS One. 6(2), e17299 (2011). 21387008 10.1371/journal.pone.0017299 PMC3046155

[b18] Glynn P. Axonal degeneration and neuropathy target esterase. Arh. Hig. Rada Toksikol. 58(3), 355–358 (2007). 18050888 10.2478/v10004-007-0029-z

[b19] Moretto A., Lotti M., Sabri M. I. & Spencer P. S. Progressive deficit of retrograde axonal-transport is associated with the pathogenesis of di-n-butyl dichlorvos axonopathy. J. Neurochem. 49(5), 1515–1522 (1987). 2444671 10.1111/j.1471-4159.1987.tb01022.x

[b20] Kretzschmar D., Hasan G., Sharma S., Heisenberg M. & Benzer S. The swiss cheese mutant causes glial hyperwrapping and brain degeneration in *Drosophila*. J. Neurosci. 17(19), 7425–7432 (1997). 9295388 10.1523/JNEUROSCI.17-19-07425.1997 PMC6573436

[b21] Emerick G. L., DeOliveira G. H., dos Santos A. C. & Ehrich M. Mechanisms for consideration for intervention in the development of organophosphorus-induced delayed neuropathy. Chem. Biol. Interact. 199(3), 177–184 (2012). 22819951 10.1016/j.cbi.2012.07.002

[b22] Morgan N. V. . PLA2G6, encoding a phospholipase A(2), is mutated in neurodegenerative disorders with high brain iron. Nat. Genet. 38(7), 752–754 (2006). 16783378 10.1038/ng1826 PMC2117328

[b23] Hall S. M. The effect of injections of lysophosphatidyl choline into white matter of the adult mouse spinal cord. J. Cell Sci. 10(2), 535–546 (1972). 5018033 10.1242/jcs.10.2.535

[b24] Jean I., Allamargot C., Barthelaix-Pouplard A. & Fressinaud C. Axonal lesions and PDGF-enhanced remyelination in the rat corpus callosum after lysolecithin demyelination. Neuroreport. 13(5), 627–631 (2002). 11973459 10.1097/00001756-200204160-00018

[b25] Read D. J., Li Y., Chao M. V., Cavanagh J. B. & Glynn P. Neuropathy target esterase is required for adult vertebrate axon maintenance. J. Neurosci. 29(37), 11594–11600 (2009). 19759306 10.1523/JNEUROSCI.3007-09.2009 PMC3849655

[b26] Hou W. Y., Long D. X., Wang H. P., Wang Q. & Wu Y. J. The homeostasis of phosphatidylcholine and lysophosphatidylcholine was not disrupted during tri-*o*-cresyl phosphate-induced delayed neurotoxicity in hens. Toxicology. 252 **(1–3)**, 56–63 (2008). 18755237 10.1016/j.tox.2008.07.061

[b27] Hermansson M., Hokynar K. & Somerharju P. Mechanisms of glycerophospholipid homeostasis in mammalian cells. Prog. Lipid Res. 50(3), 240–257 (2011). 21382416 10.1016/j.plipres.2011.02.004

[b28] Chakraborty M. & Jiang X. C. Sphingomyelin and its role in cellular signaling. Adv. Exp. Med. Biol. 991, 1–14 (2013). 23775687 10.1007/978-94-007-6331-9_1

[b29] Husain K., Ansari R. A. & Ferder L. Pharmacological agents in the prophylaxis/treatment of organophosphorous pesticide intoxication. Indian J. Exp. Biol. 48(7), 642–650 (2010). 20929049

[b30] Pope C. N. & Padilla S. Potentiation of organophosphorus-induced delayed neurotoxicity by phenylmethylsulfonyl fluoride. J. Toxicol. Env. Health. 31(4), 261–273 (1990). 2254952 10.1080/15287399009531455

[b31] Kayyali U. S., Moore T. B., Randall J. C. & Richardson R. J. Neurotoxic esterase (NTE) assay - Optimized conditions based on detergent-Induced shifts in the phenol/4-aminoantipyrine chromophore spectrum. J. Anal. Toxicol. 15(2), 86–89 (1991). 2051750 10.1093/jat/15.2.86

[b32] Cox B. & Emili A. Tissue subcellular fractionation and protein extraction for use in mass-spectrometry-based proteomics. Nat. Protoc. 1(4), 1872–1878 (2006). 17487171 10.1038/nprot.2006.273

[b33] Folch J., Lees M. & Stanley G. H. S. A simple method for the isolation and purification of total lipides from animal tissues. J. Biol. Chem. 226(1), 497–509 (1957). 13428781

[b34] Nie H. G. . Lipid profiling of rat peritoneal surface layers by online normal- and reversed-phase 2D LC QToF-MS. J. Lipid Res. 51(9), 2833–2844 (2010). 20526000 10.1194/jlr.D007567 PMC2918466

